# Supplementary material for: Splice-Junction-Based Mapping of Alternative Isoforms in the Human Proteome
Source: Cell Rep. Author manuscript; Available in PMC 2020 Jan 15. (PMC6961840; doi:10.1016/j.celrep.2019.11.026)
Supplement: 2 [file NIHMS1546469-supplement-2.zip › DF1/PXD010154/Heart/Heart_79_EHMT2_LVEEENICLHWASFTGSAAIAEVLLNARCDLHAVNYHGDTP.pdf]

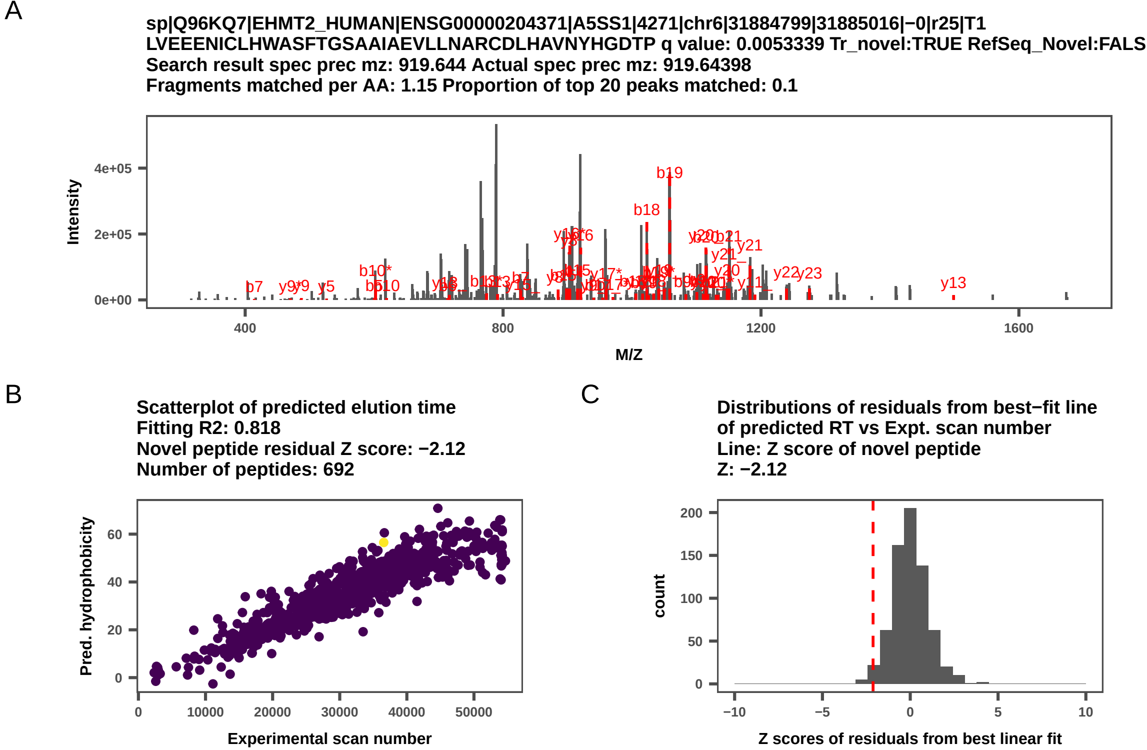
A
sp|Q96KQ7|EHMT2_HUMAN|ENSG00000204371|A5SS1|4271|chr6|31884799|31885016|−0|r25|T1
LVEEENICLHWASFTGSAAIAEVLLNARCDLHAVNYHGDTP q value: 0.0053339 Tr_novel:TRUE RefSeq_Novel:FALS
Search result spec prec mz: 919.644 Actual spec prec mz: 919.64398
Fragments matched per AA: 1.15 Proportion of top 20 peaks matched: 0.1
Intensity
4e+05
2e+05
0e+00
b19
b18
b7
y5
y13
y22
y23
y21
400
800
1200
1600
M/Z
B
Scatterplot of predicted elution time
Fitting R2: 0.818
Novel peptide residual Z score: −2.12
Number of peptides: 692
Pred. hydrophobicity
60
40
20
0
10000
20000
30000
40000
50000
Experimental scan number
C
Distributions of residuals from best−fit line
of predicted RT vs Expt. scan number
Line: Z score of novel peptide
Z: −2.12
count
200
150
100
50
0
−10
−5
0
5
10
Z scores of residuals from best linear fit
